# Dynamic networks of intrinsically disordered regions in nuclear proteins

**DOI:** 10.2142/biophysico.bppb-v23.0020

**Published:** 2026-06-06

**Authors:** Yoshifumi Nishimura

**Affiliations:** 1 Graduate School of Medical Life Science, Yokohama City University, Yokohama, Kanagawa 230-0045, Japan

**Keywords:** nucleosome, NMR, phase separation, heterochromatin protein 1, histone chaperone

## Abstract

Chromatin in the eukaryotic nucleus is organized into transcriptionally active euchromatin and transcriptionally silent heterochromatin. Its fundamental unit, the nucleosome, consists of two copies each of histones H2A, H2B, H3, and H4 wrapped by ~145 base pairs of DNA, flanked by linker-DNA that binds linker-histone H1 to form a chromatosome. In euchromatin, histone N-terminal tails (N-tails) are extensively acetylated. In facultative heterochromatin, H3K27 is methylated (H3K27me) and the H2A C-terminal tail (C-tail) is ubiquitinated (H2Aub), whereas in constitutive heterochromatin, H3K9 is methylated (H3K9me) and recognized by heterochromatin protein 1 (HP1). The N-tail of the HP1α homologue is phosphorylated, enhancing its binding to H3K9me and promoting liquid–liquid phase separation (LLPS). In addition, the C-tails of the histone chaperones FACT and NAP1 mediate binding to H2A-H2B. These N-tails and C-tails are intrinsically disordered regions (IDRs), whose dynamic conformations are accessible primarily through NMR spectroscopy. NMR has revealed dynamic IDR interaction networks essential for chromatin regulation, including H3 N-tail acetylation enhanced by H4 N-tail acetylation on linker-DNA, its suppression by H1, H3K27 methylation promoted by H2Aub, H3 N-tail acetylation induced by phosphorylated FACT C-tail, the role of the NAP1 C-tail in H2A-H2B binding, and phosphorylation-dependent enhancement of HP1 N-tail binding to H3K9me and LLPS. Thus, NMR has illuminated how dynamic interactions among nuclear IDRs play central roles in establishing euchromatin and heterochromatin, providing insights into the molecular basis of nuclear function.

## Significance

Chromatin architecture is essential for understanding cell type–specific and cancer-specific regulation. Numerous modifications within intrinsically disordered regions (IDRs) of nuclear proteins critically influence this architecture, yet the dynamic structures of most IDRs remain elusive. Recent NMR studies have revealed modification-dependent IDR dynamics and interaction networks relevant to chromatin regulation, including the effects of histone tail acetylation, methylation, and ubiquitination; phosphorylation of the HP1 tail that enhances binding to methylated histones and promotes LLPS; modulation of histone-tail dynamics by FACT tail phosphorylation; and NAP1-tail binding to H2A–H2B.

## Introduction

The eukaryotic genome is packaged into chromatin, a highly organized and dynamic structure. Chromatin architecture plays a central role in regulating transcription, replication, and genome stability. Recent integrative genomic and imaging studies have revealed that the nucleus is partitioned into spatially segregated chromatin domains known as the A and B compartments [1–4]. A compartment corresponding to euchromatin comprises transcriptionally active, early-replicating regions, whereas B compartment corresponding to heterochromatin contains transcriptionally silent, late-replicating regions. These higher order chromatin compartments establish cell type-specific gene expression programs. Humans possess more than 250 distinct cell types, each defined by characteristic transcriptional programs associated with specific A-compartment regions. Each cell contains two copies of the 3-billion–base-pair haploid genome distributed across 24 chromosomes, yielding approximately 6 billion base pairs of DNA—roughly 2 meters in length—compactly organized within 48 chromosomes.

**Figure 1:**
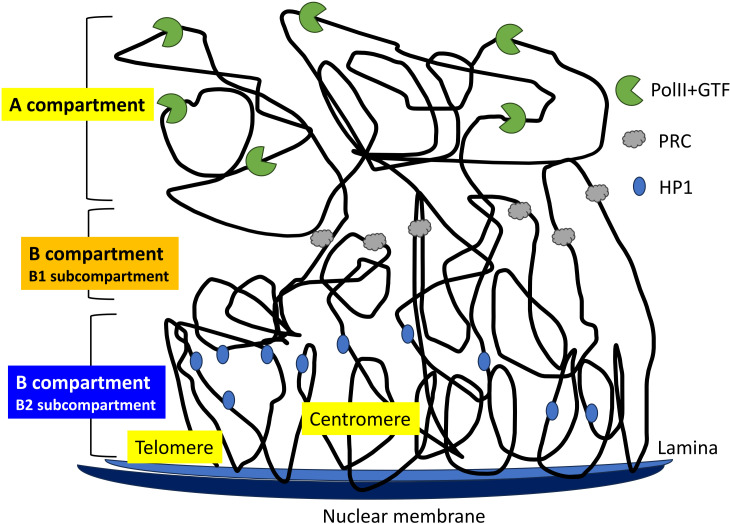
Schematic representation of A and B compartments in eukaryotic nucleus [3,4].

Figure 1 shows a schematic representation of the nuclear compartments [3,4]. Within the A compartment, transcriptionally active regions are micropatterned as clusters of RNA polymerase II (Pol II) formed through localized condensation at super-enhancers bound by specific transcription factors [3–6]. The B compartment is further subdivided into the B1 sub-compartment, which represents facultative heterochromatin capable of transitioning between active and inactive states in a cell type– and differentiation–dependent manner through regulation by Polycomb repressive complexes 1 (PRC1) and 2 (PRC2), and the B2 sub-compartments, which form constitutive heterochromatin anchored to the nuclear lamina and heterochromatin protein 1 (HP1), remaining stably repressed across most cell types [3,4]. Typical constitutive heterochromatin includes centromeres, which are formed by the chromosome passenger complex (CPC)—a large assembly of proteins that localize to the centromere during specific stages of the cell cycle, including inner centromere protein (INCENP) [7,8]—and telomeres, which consist of the shelterin complex, including telomere repeat–binding factors 1 (TRF1) and 2 (TRF2), both of which bind to telomeric DNA composed of TTAGGG repeat duplexes with a 3' overhang single stranded DNA [9].

The fundamental structural unit of chromatin is the nucleosome core particle (NCP), comprising two copies each of the four core-histones (H2A, H2B, H3, and H4) wrapped by approximately 146 base pairs (bp) of core-DNA, flanked by several lengths of linker-DNA forming the nucleosome [10–12]. Binding linker-histone H1 to the linker-DNA generates chromatosome, which contributes to higher-order chromatin architecture. These histones contain flexible tail regions: the N-terminal tails (N-tails) of H2A, H2B, H3, H4, and H1, and the C-terminal tails (C-tails) of H2A and H1. These flexible tails are specifically post-translationally modified by acetylation, phosphorylation, methylation, and ubiquitination [13–15]. These modifications are strongly coupled with the formation of chromatin architecture. Furthermore, during transcription, replication, and DNA repair chromatin remodeling factors alter the chromatin structure and several histone chaperones—such as FACT (facilitates chromatin transcription) and NAP1 (nucleosome assembly protein 1)—facilitate the eviction and/or deposition of histones into nucleosomes for example [16].

Recent advances in cryogenic electron microscopy (cryo-EM) and X-ray crystallography have elucidated the rigid tertiary structures of numerous chromatin-associated protein complexes, including super-enhance complexes, the pre-initiation complex consisting of Pol II and general transcription factors (GTFs), and transcribing Pol II on nucleosomes in association with histone chaperones [6,17]. In cells, however, the dynamic architecture of these complexes is governed by the coordinated behavior of many modular building blocks—DNA, RNA, and protein assemblies—whose rearrangements proceed through cell type-specific and time-dependent steps. Cryo-EM tomography captures these stepwise cellular dynamics as successive snapshots during time-dependent cellular responses [2]. In this sense, a cell can be viewed as an automobile moving along a road composed of numerous mechanical components that interact to ensure proper function.

**Figure 2:**
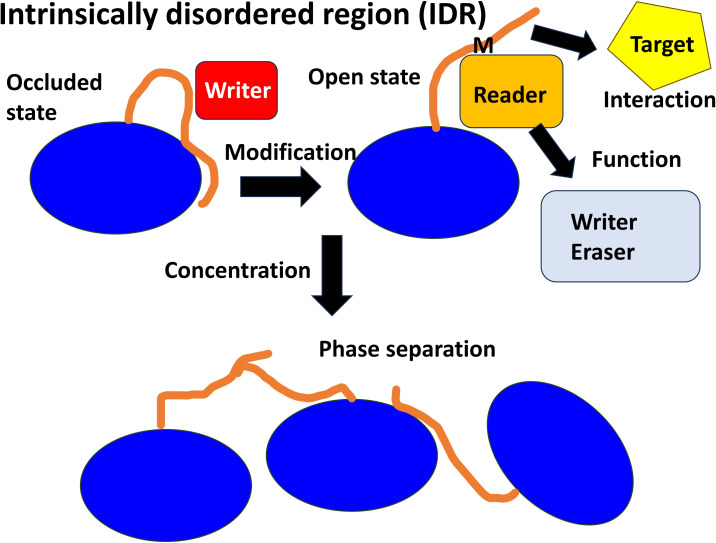
Schematic representation of a protein consisting of a core domain (blue ellipse) with an IDR (orange line).

However, a cell does not consist of rigid structural units like mechanical compartments; instead, it is composed of materials that include not only rigid domains but also flexible tails and hinges, known as intrinsically disordered regions (IDRs) [18–23]. Typical nuclear proteins contain both structured domains and IDRs located at the N- or C-terminal tails or at hinge regions between domains. Figure 2 illustrates a protein composed of a rigid domain and a flexible, string-like IDR. IDRs dynamically interact with their own domains (occluded state) and/or protrude outward (open state). This dynamic equilibrium is commonly regulated by post-translational modifications such as phosphorylation, ubiquitination, methylation, and acetylation, because the flexible IDR serves as an accessible substrate for modifying enzymes (writers). In the open state, the protein can interact with other target proteins, and the modified IDR can be recognized by reader proteins, which in turn function as writers or erasers to modify additional proteins. When concentrated, IDRs can also engage in intermolecular interactions with other IDRs and/or domains to induce liquid–liquid phase separation (LLPS), forming droplets [23–30]. Many chromatin-associated factors—including nucleosomes, HP1, and histone chaperones—undergo LLPS and are thought to contribute to the formation of A and B compartments.

IDRs remain largely inaccessible to conventional structural analyses such as X-ray crystallography and cryo-EM. In contrast, nuclear magnetic resonance (NMR) spectroscopy, in combination with molecular dynamics (MD) simulations, has begun to illuminate the dynamic behavior of histone tails within nucleosomes and histone chaperones, as well as the dynamic architecture of the general transcription factor TFIIH complexes [31–33]. Moreover, NMR has revealed the LLPS mechanism of HP1 that underlies heterochromatin formation.

## Histone tails

### Concept of reader and writer/eraser for histone tails

The histones contain the N-terminal tails (N-tails) of H2A, H2B, H3, and H4, as well as the C-terminal tail (C-tail) of H2A, which are specifically post-translationally modified by acetylation, phosphorylation, methylation, and ubiquitination [13–15]. The relationship between chromatin architecture and histone modifications has been extensively studied as a central aspect of epigenetic regulation. For example, in euchromatin or the A compartment, lysine residues in the N-tails are highly acetylated, and the lysine at the fourth position of the H3 N-tail is methylated (H3K4me). In contrast, in heterochromatin or the B compartment, the N-tails are deacetylated. In constitutive heterochromatin (the B2 sub-compartment), the lysine at the ninth position of the H3 N-tail is methylated (H3K9me), whereas in facultative heterochromatin (the B1 sub-compartment), the lysine at the 27th position of the H3 N-tail is methylated (H3K27me), and the H2A C-tail is ubiquitinated [3,4].

These modifications contribute to networks of chromatin-associated proteins that function as readers and/or writers/erasers. For example, acetylated lysine residues in the H4 N-tail are recognized by the bromodomain (reader) of the histone acetyltransferase (HAT) GCN5, whose catalytic core domain (writer) acetylates the H3 N-tail to promote euchromatin formation. In contrast, during heterochromatin formation or in the B compartment, the N-tails of H3 and H4 are deacetylated by histone deacetylases (HDACs; erasers), which often act together with factors such as H3K4 demethylases (erasers) and H3K9 methyltransferases (writers) to recruit HP1 (reader). In facultative heterochromatin, H3K27 is methylated by PRC2 (writer), which binds to ubiquitinated H2A C-tails as a reader, while PRC1 (writer) ubiquitinates the H2A C-tail; conversely, PRC1 also binds to methylated H3K27 as a reader [3]. These combinations of readers and writers/erasers establish regulatory networks within chromatin that ensure the proper formation of A and B compartments in the nucleus.

### Additional concept: Mutual correlations between histone tails

To date, many tertiary structures of nucleosomes have been determined by X-ray crystallography and cryo-EM. Numerous complex structures of chromatin-related proteins bound to nucleosomes have also been solved, revealing that the essential core architecture of the nucleosome is highly conserved. However, in almost all cases, the histone tails are not visualized because of their intrinsic flexibility. In contrast, NMR signals are detected almost exclusively from histone tails, as these flexible, string-like regions behave like small molecules with rapid motions that yield sharp NMR peaks, whereas the histone cores have high molecular weight and slow motions, producing extremely broad signals buried in noise [34–37].

**Figure 3:**
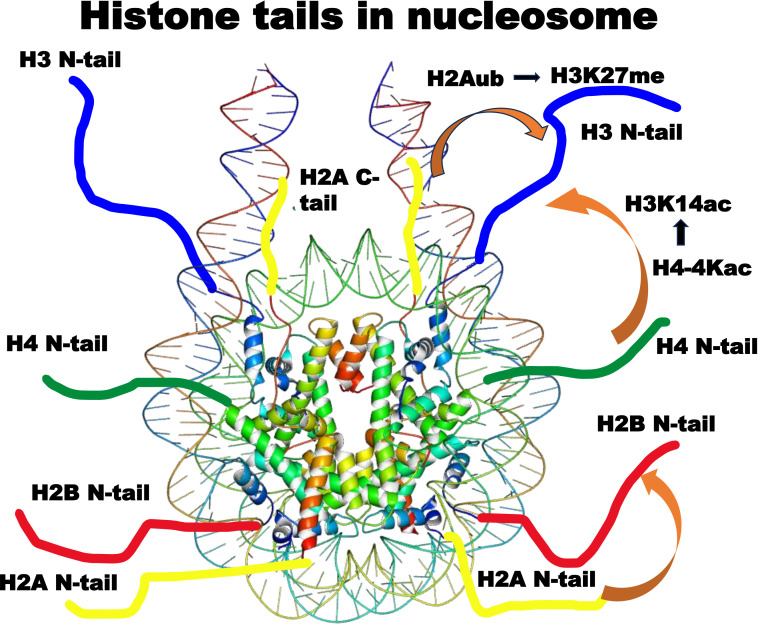
Schematic representation of histone tails in a nucleosome. The nucleosome core structure is taken from the H1.8 bound nucleosome in Xenopus egg extract observed by cryo-EM [PDB ID: 7KBF].

**Figure 4:**
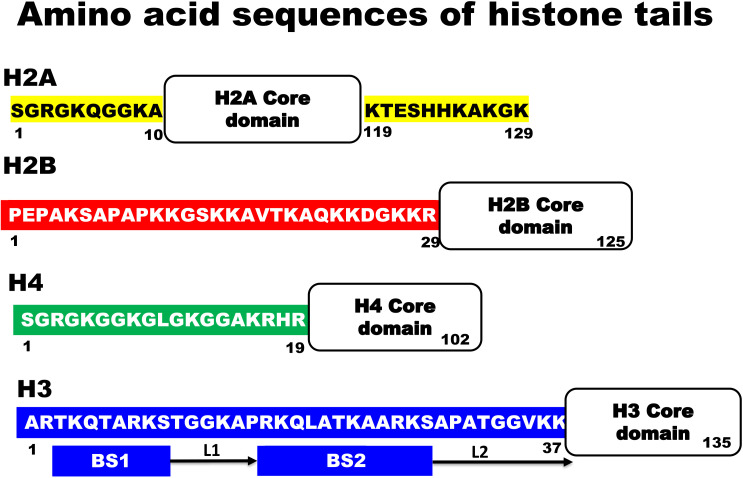
Amino acid sequences of histone tails. Almost all these amide protons except proline residues have been identified by ^1^H-^15^N NMR.

Figure 3 presents a schematic representation of histone tails protruding from the nucleosome structure [PDB ID: 7KBF], illustrating that dynamic interactions between tails are plausible: between the H3 N-tail and the H2A C-tail in a linker-DNA–dependent manner; between the H2A and H2B N-tails independently of linker-DNA; and between the H4 and H3 N-tails in a linker-DNA–dependent manner [32,33,38–41]. Most of these interactions cannot be detected by cryo-EM or X-ray crystallography. To examine the dependence of histone tail dynamics on linker-DNA, our group prepared NCPs wrapped by 145-bp DNA lacking linker-DNA, as well as nucleosomes containing 193-bp DNA with 24-bp linker-DNA at both ends. Using these samples together with specifically labeled histones (H2A, H2B, H3, and/or H4), our group analyzed the dynamics of these histone tails by two-dimensional (2D) ^1^H-^15^N heteronuclear single-quantum coherence (HSQC) spectroscopy (Figure 4) [32,33,38–41].

### H3 N-tail dynamics in the nucleosome and the NCP

**Figure 5:**
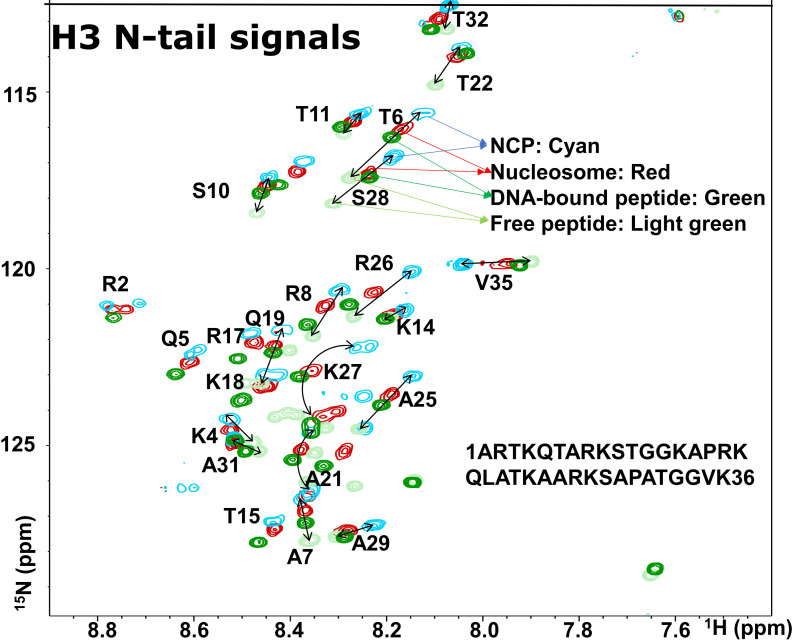
H3 N-tail amide signals of the isolated N-tail peptide (A1-K36: light green), the peptide bound to DNA (green), nucleosome (red), and NCP (cyan) by ^1^H-^15^N HSQC spectra modified from Supplementary Figure S8 of Ref. [40].

Using ^15^N-labeled H3 and unlabeled H2A, H2B, and H4, two-dimensional ^1^H-^15^N NMR spectra have been reported for both the nucleosome and the NCP, along with spectra of the ^15^N-labeled H3 N-tail peptide in its free and DNA-bound states [36–40]. As shown in Figure 5, almost all H3 N-tail signals in the NCP exhibit a slight but significant high-field shift relative to the corresponding signals in the nucleosome under identical conditions [39,40]. Nearly all nucleosome signals lie very close to those of the DNA-bound H3 N-tail peptide and far from those of the free-peptide signals, whereas the NCP signals shift even further away from the nucleosome signals in both the ^1^H and ^15^N dimensions [39,40]. The dynamic motion of the H3 N-tail in the nucleosome, as well as its accessibility to histone-modifying enzymes, is suppressed compared with the isolated H3 N-tail peptide [36]. MD simulations further showed that the H3 N-tail in the NCP dynamically and robustly packs onto core-DNA, producing NMR signals similar to those of the isolated H3 N-tail peptide bound to an H3-tailless NCP [37]. The two basic segments, BS1 (T3–K9) and BS2 (R17–S28) (Figure 4), rather than the loop regions L1 (S10–P16) and L2 (A29–K36), appear to constitute the primary DNA-binding regions through electrostatic interactions [36–40], as evidenced by their pronounced salt-dependent chemical shift changes [38–40].

**Figure 6:**
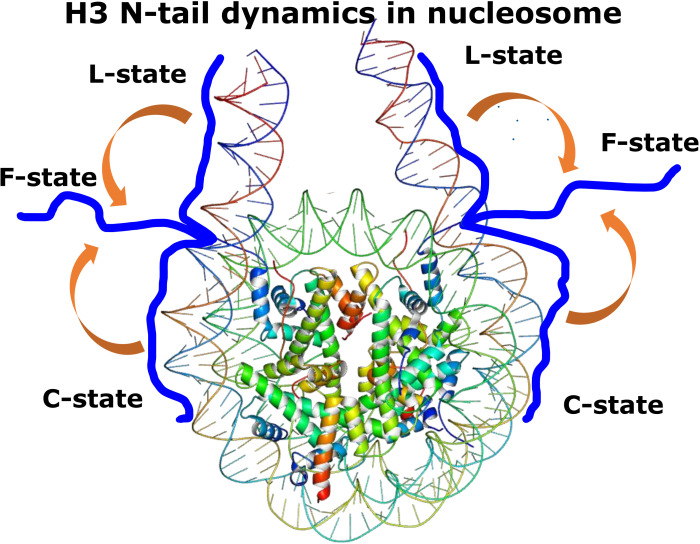
Schematic representation of the H3 N-tail dynamics in the nucleosome fluctuating among three states: a linker-DNA contact state (L-state), a cored-DNA bound state (C-state), and a free unbound state (F-state).

These observations suggest that the H3 N-tail in the nucleosome dynamically fluctuates among three states: a linker-DNA contact state (L-state), a core-DNA contact state (C-state), and a free unbound state (F-state), as illustrated in Figure 6. In the absence of linker-DNA, as in the NCP, the H3 N-tail dynamically exchanges between the C- and F-states [32,33,38–40].

Considering the direction of the chemical-shift changes upon DNA binding, the affinity of the H3 N-tail for DNA appears to be higher in the C-state than in the L-state [32,33,39,40]. This may reflect differences in duplex-DNA density: the H3 N-tail protruding between two core-DNA gyres can interact with both duplexes, whereas in the linker region it contacts only a single duplex. In addition, heteronuclear NOE (nuclear Overhauser effect) measurements show that the dynamic motion of the H3 N-tail is more restricted in the NCP than in the nucleosome [39]. The affinity of the L-state resembles the nonspecific binding of the isolated H3 N-tail peptide to DNA. In the nucleosome, the H3 N-tail appears to adopt the L-state predominantly rather than the presumed high-affinity C-state, likely because linker-DNA is highly flexible whereas core-DNA is not. X-ray and cryo-EM structures of the nucleosome consistently reveal well-defined core-DNA but not linker-DNA, reflecting their distinct flexibilities. Thus, entropic effects likely favor H3 N-tail binding to linker-DNA over core-DNA. These distinct L- and C-states correlate well with the markedly different acetylation rates catalyzed by the HAT domain of Gcn5: under identical conditions, the acetylation rate in the nucleosome is approximately 4.3×10^–5^ s^–1^, whereas in the NCP it is much slower, approximately 6.9×10^–6^ s^–1^ [39].

### Dynamic correlation between H3 and H4 N-tails

The H4 N-tail exhibits similar NMR signals in both the NCP and the nucleosome, indicating that the H4 N-tail dynamically contacts core-DNA independently of linker-DNA [39]. Although salt-induced chemical-shift changes of the H4 N-tail are modest, the signal intensities increase markedly [39]. In the NCP, the H4 N-tail shows small chemical-shift changes but reduced conformational flexibility compared with the free H4 N-tail peptide; together with MD simulations, these findings indicate that the H4 N-tail forms a fuzzy interaction with core-DNA [42]. This conclusion is further supported by NMR measurements of paramagnetic relaxation enhancements (PREs) combined with MD simulations [43]. The distinct behaviors of the H3 and H4 N-tails likely originate from their structural orientations: the H3 N-tail protrudes between the two gyres of core-DNA, whereas the H4 N-tail protrudes above or below the core-DNA.

Using tetra-acetylated H4 at K5, K8, K12, and K16 (H4-4Kac), the chemical-shift changes of the H4 N-tail—except at the acetylated lysine residues—were found to be modest, but the signal intensities increased in both acetylated NCP and nucleosome, suggesting that the H4-4Kac N-tail is released from the core-DNA contact state [38,39]. Nevertheless, the core structure of the H4-4Kac NCP is nearly identical to that of the conventional NCP [45]. This interpretation is supported by recent MD simulations on conventional and H4-4Kac nucleosomes [44]. H4-4Kac induces substantial signal changes in the H3 N-tail in the nucleosome but not in the NCP [38,39]. The H4-4Kac nucleosome exhibits paired major and minor signals for H3 N-tail residues in BS1, BS2, and L2 [38]. For most of these residues, the major signal lies between the corresponding NCP and nucleosome signals, indicating that the H3 N-tail population shifts toward the C-state. In contrast, the minor signal lies between the nucleosome and free-peptide signals, suggesting a shift toward the F-state. Thus, H4-4Kac appears to reduce the population of the H3 N-tail L-state while increasing both the C-and F-states. The H3 N-tail in the H4-4Kac nucleosome seems to adopt two distinct conformations in the NMR time scale. Owing to the formation of new dynamic conformations or an increased F-state population, H4-4Kac markedly enhances H3K14 acetylation by the GCN5 core enzyme by approximately twofold, from 4.1×10^–5^ s^–1^ to 8.1×10^–5^ s^–1^ [38]. These mutual dynamic correlations between the H3 and H4 N-tails likely contribute to the promotion of euchromatin formation.

### Dynamic correlation of linker-histone H1 tails with H3 and H4 N-tails

**Figure 7:**
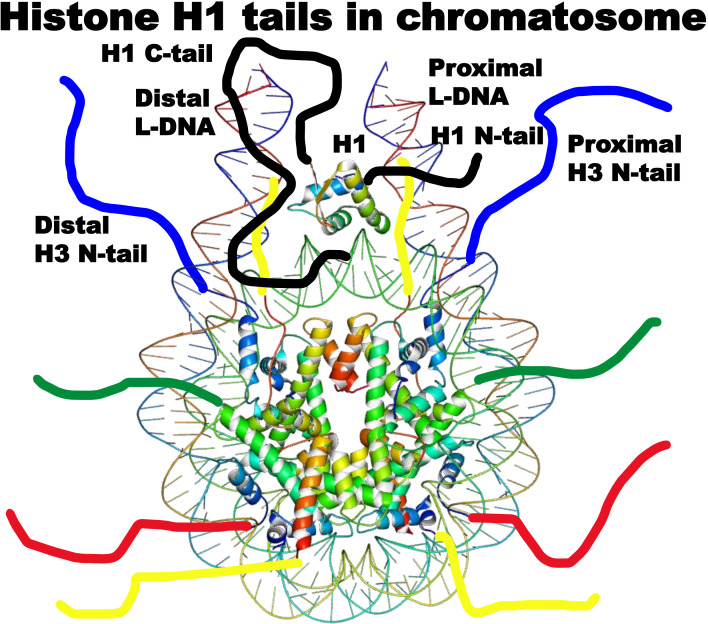
Schematic representation of the H1 and H3 tail dynamics in chromatosome. Yellow lines are N- and C-tails of H2A. Red and green lines are N-tails of H2B and H4, respectively, as in Figure 3.

Figure 7 shows a schematic representation of the chromatosome. Linker-histone H1 binds to the proximal linker-DNA at the dyad axis of the core-DNA and contains short N-terminal and long C-terminal tails [46–48]. In the human somatic isoform H1.4, which is implicated in gene regulation, the globular domain comprises residues 35-108, flanked by the N-tail (residues 1–34) and the C-tail (residues 109–218). Cryo-EM structures have shown that the H1 C-tail primarily binds to distal linker-DNA [48], whereas coarse-grained MD (CGMD) simulations indicate that the H1 C-tail is sufficiently long to dynamically associate not only with distal linker-DNA but also with dyad core-DNA and proximal linker-DNA [44,49,50].

Upon addition of H1.4 to the nucleosome, nearly all NMR signals of the H3 N-tail shift toward NCP-like positions, indicating that the L-state is suppressed by H1.4 binding; apparently, the H1 N-tail inhibits the H3 N-tail binding to linker-DNA [39]. Interestingly, residues in BS1 exhibit both NCP-like and nucleosome-like signals. These observations suggest that linker-histone H1.4 induces asymmetric conformations of the two H3 N-tails, at least within the BS1 region: one H3 N-tail dynamically and robustly adopts the C-state, reducing its accessibility to the enzyme, whereas the other remains in the L-state and thus remains accessible. Nevertheless, the dynamic equilibrium between the C- and L-states occurs on the timescale of the enzymatic reaction, such that the acetylation rate of H3K14 by Gcn5 in the chromatosome (approximately 1.7×10^–5^ s^–1^) is only slightly slower than that in the nucleosome (4.3×10^–5^ s^–1^) [39,44]. This indicates that linker-histone H1 does not repress H3 N-tail acetylation on its own. Because linker-histone H1 dynamically fluctuates between the nucleosome-bound and released state on a timescale of several minutes, the asymmetric structure of the H3 N-tail in the chromatosome effectively behaves symmetrically on the timescale of the enzyme reaction [39].

The effect of H4-4Kac on the chromatosome was then examined by cryo-EM, CGMD, and NMR [44]. Cryo-EM revealed that the H4-4Kac chromatosome retains essentially the same core histone and core-DNA structures as the conventional chromatosome [48]. CGMD simulations indicated that H4 N-tail is released from core-DNA binding in H4-4Kac chromatosome, whereas the H3 N-tail exhibits similar dynamics in both chromatosomes. However, NMR showed that the H3 N-tail signals of the H4-4Kac chromatosome were essentially identical to NCP-like signals, with no nucleosome-like signals even in the BS1 region. This indicates that in the H4-4Kac chromatosome, both H3 N-tails predominantly adopt a dynamically robust C-state, distinct from the unmodified chromatosome. Consequently, the acetylation rate of the H3 N-tail is markedly reduced in the H4-4Kac chromatosome to ~4.5×10^–6^ s^–1^, compared with ~1.7×10^–5^ s^–1^ in the conventional chromatosome [44]. These findings suggest that linker-histone H1 does not inhibit H3 N-tail acetylation by itself, but rather suppresses the enhancement of H3 N-tail acetylation that is otherwise promoted by H4 N-tail acetylation. Thus, linker-histone H1 appears to inhibit the cooperative acetylation of the H3 and H4 N-tails, contributing to the maintenance of heterochromatin [44].

### Dynamic correlation between the H3 N-tail and the H2A C-tail

For facultative heterochromatin, PRC1 and PRC2 play central roles in epigenetic gene regulation. Mono-ubiquitination of histone H2AK119 (H2AK119ub) by PRC1 promotes PRC2-mediated trimethylation of H3K27 (H3K27me3) on nucleosomes [3]. In the NCP, the H3 N-tail predominantly adopts the C-state, which suppresses H3K27 methylation. Although H2AK119ub NCP exhibits similar H3 N-tail dynamics, maintaining its DNA-contacting conformation, H2AK119ub enhances H3K27 methylation by enabling PRC2 and its cofactors to bind the ubiquitin moiety on H2A [40].

In the unmodified nucleosome, the H3 N-tail dynamically adopts the L-state, which enhances H3K27 methylation to a level comparable to that observed for the H2AK119ub NCP. Cryo-EM analysis revealed that the H2AK119ub nucleosome retains essentially the same core structure as the unmodified nucleosome, aside from the additional ubiquitin moiety on the H2A C-tail. However, nearly all H3 N-tail NMR signals shift to positions between those of the unmodified nucleosome and the NCP, suggesting strengthened DNA contact and the formation of a specific dynamic conformation [40]. Both the H3 N-tail dynamic shift and the presence of the H2AK119ub moiety synergistically enhance PRC2-mediated H3K27 methylation approximately twofold compared with the unmodified nucleosome [40].

### Dynamic correlation between H2A and H2B N-tails

Using ^2^H/15N- or ^2^H/^13^C/^15^N-labeled H2A and H2B in both the NCP and nucleosome, our group extensively analyzed the H2A N-tail (S1–G8), H2A C-tail (T120–K129), and H2B N-tail (E2–K27) by NMR, including relaxation measurements and hydrogen-exchange rates under different salt conditions [41]. These data were compared with those of isolated H2A and H2B tail peptides, both free and DNA-bound, as well as with the H2A-H2B heterodimer. In addition, temperature-dependent chemical-shift changes of the tails were examined. Chemical-shift indices for backbone Cα and Cβ atoms indicated that the H2A N- and C-tails and the H2B N-tail adopt random-coil conformations without detectable secondary structure in both the NCP and nucleosome.

As expected, the H2A C-tail exhibited distinct chemical shifts between the NCP and nucleosome, with clear differences in conformational-exchange rates depending on the presence of linker-DNA, although local fluctuation amplitudes and hydrogen-exchange rates were similar. In the nucleosome, the H2A C-tail likely interacts with linker-DNA, and this dynamic interaction persists even at high salt concentrations, suggesting that the H2A C-tail fluctuates between the two linker-DNA arms. In the NCP, the H2A C-tail appears to interact dynamically with core-DNA [41].

Both the H2A and H2B N-tails showed essentially identical chemical shifts in the NCP and nucleosome, independent of linker-DNA. However, both tails exhibited doublet signals at 25 mM KCl, but not at 400 mM KCl, indicating the presence of two distinct DNA-contact conformations, as inferred by comparison with the chemical shifts of free and DNA-bound tail peptides. These doublet signals persisted even when the core-DNA sequence was changed from the commonly used asymmetric 601 sequence to a symmetric sequence [41].

For the H2A N-tail, the doublet signals appear to correspond to strong and weak DNA-contact states. The strong-contact state shows low motional amplitudes and high conformational-exchange rates, likely reflecting polymorphic DNA interactions, whereas the weak-contact state shows high motional amplitudes and low exchange rates. At high salt, the H2A N-tail exhibits higher motional amplitudes and reduced conformational exchange, resembling the DNA-free peptide. The presence of two contact states, or the weak-contact conformation of H2A, may arise from inhibition of DNA contact by the H2B N-tail. The H2B N-tail doublet signals showed no major differences in dynamics but displayed significant differences in chemical shifts and in temperature-dependent chemical-shift deviations, suggesting that the two components correspond to different types of DNA-contact states [41].

The H2B N-tail protrudes between the two core-DNA gyres and dynamically contacts two duplexes from different sides—the entry/exit side or the opposite side—whereas the H2A N-tail protrudes above or below the core-DNA and contacts either the major or minor groove. These two different DNA contact modes of H2A and H2B likely correspond to two sets of NMR signals observed for each tail. MD simulations showed that the H2A minor-groove conformation is observed when the H2B N-tail is positioned on the opposite side, whereas when the H2B N-tail occupies the entry/exit side, the H2A N-tail adopts both major- and minor-groove conformations. Together, the NMR data and MD simulations suggest that the minor-groove conformation of the H2A N-tail contact to DNA more strongly than the major-groove conformation, and that when the H2B N-tail occupies the entry/exit side, the H2A N-tail shifts its DNA contact toward the major groove, partially away from the minor groove [41].

## IDRs in histone chaperones for recruiting histone H2A-H2B

To initiate nucleosome formation, one (H3-H4)_2_ tetramer first binds to DNA to form a tetrasome. An H2A-H2B heterodimer is then deposited onto the tetrasome to generate a hexasome, followed by the incorporation of a second H2A-H2B heterodimer to complete the octasome, or nucleosome. Histone chaperones are essential for these processes, as they facilitate the binding of both the H2A-H2B heterodimer and the (H3-H4)_2_ tetramer. During transcription and replication, histone chaperones such as FACT and NAP1 play key roles in the eviction and deposition of H2A-H2B [16,17]. Notably, both chaperones contain long C-terminal tails or IDRs that mediate their interactions with the H2A-H2B heterodimer.

### Dynamic correlations of histone chaperone FACT and histone tails

The histone chaperone FACT is an essential factor that promote nucleosome disassembly and assembly by evicting or depositing an H2A-H2B heterodimer and by facilitating partial DNA unravelling during transcription [16,17]. FACT consists of two subunits, SSRP1 and SPT16. SPT16 contains a C-terminal acidic intrinsically disordered region (AID) of 38 amino acids, which induces structural alterations during nucleosome unwrapping [51]. In the NCP, the terminal portion of the 145-bp DNA near the entry/exit site is displaced from the histone core by the phosphorylated AID (pAID) of SPT16. As a model of an unwrapped NCP bound to FACT, a complex was prepared in which 112-bp DNA and the pAID segment of SPT16 are jointly wrapped around the histone core instead of the conventional 145-bp DNA. Cryo-EM analysis revealed that, in place of the missing 33-bp DNA segment, the pAID segment asymmetrically wraps around the exposed DNA-binding surfaces of H3, H2A, and H2B in the 112-bp nucleosome [52]. Approximately three-quarters of the particle resembles the canonical NCP, while the remaining quarter is wrapped by pAID and DNA, generating an asymmetric NCP-like structure.

Reflecting this asymmetry, the H3 N-tail exhibits distinct doublet signals: one corresponding to the canonical NCP state and the other corresponding to the proximal pAID-associated state [53]. When the pAID segment is replaced by the 33-bp DNA to reconstitute a canonical NCP—with a gap between the 33-bp DNA and 112-bp DNA fragments—the H3 N-tail signals become identical to those of the canonical NCP; the pAID-side signals disappear completely, leaving only the NCP-like signals. Because the two H3 N-tails can be clearly distinguished in the 112-bp DNA/pAID NCP by NMR, acetylation of H3K14 by GCN5 can be separately monitored for each tail. The pAID-side H3 N-tail is acetylated much more rapidly than the DNA-side tail or tail in the canonical NCP [53]. These findings indicate that FACT partially unwraps the NCP to facilitate H3 N-tail acetylation, suggesting that such alterations in histone-tail dynamics may play key roles in switching between heterochromatin and euchromatin.

Next, the dynamic structures of the H2A and H2B tails were examined in the 112-bp DNA/pAID NCP and in the 112-bp hexasome, which contains one (H3–H4) tetramer and one H2A-H2B dimer. Multiple conformations of the H2A and H2B tails in the 112-bp DNA/pAID NCP can be clearly distinguished as proximal (pAID-side) and distal (DNA-side) states [54]. On the pAID side, the H2A and H2B N-tails contact pAID more frequently than they contact DNA in the canonical NCP. On the DNA side, the H2B N-tail shows markedly increased DNA contact, whereas the H2A N-tail predominantly adopts a reduced-contact conformation. Thus, FACT modulates chromatin signaling and the accessibility of the H2A and H2B N-tails on both sides of the NCP, in contrast to its modulation of only the proximal H3 N-tail [54]. The alterations on the H2A and H2B N-tails both in the pAID and DNA sides likely contribute to promoting eviction of the H2AH2B dimer.

### Dynamic binding of histone chaperone NAP1 to the H2A-H2B heterodimer

For the eviction and deposition of H2A-H2B heterodimer from or onto the nucleosome, the histone chaperone NAP1 plays a crucial role [16]. NAP1 contains a dimerization domain and a C terminal acidic domain (CTAD), an IDR essential for interacting with H2A-H2B. The H2A-H2B heterodimer is a relatively small molecule of approximately 28 kDa, and its overall structure has been determined by NMR in combination with MD simulations [55]. Both H2A and H2B possess a core histone fold comprising α_1_–β_1_–α_2_–β_2_–α_3_–α_C_, together with long H2A N- (residues 1–22) and C-terminal tails (residues 101–129) and a long H2B N-tail (residues 1–35); outside the histone fold of H2A, the N-terminal α_N_ helix and the C-terminal β_3_ strand and 3_10_ helix observed in the nucleosome are entirely disordered in the H2A-H2B heterodimer [55]. This represents one of the first complete structural views of a histone complex containing full-lengths IDRs.

To obtain the crystal structure of NAP1 bound to H2A-H2B, both IDRs were deleted; for example, *C. elegans* NAP1 (ceNAP1) lacking both the N-terminal tail (residues 1–9) and C-terminal tail (residues 297–316) binds via its concave groove to a single H2A-H2B heterodimer consisting of the C-terminus of *C. elegans* H2B (residues 30–122) fused to the N-terminus of *C. elegans* H2A (residues 10–121) [56]. To investigate the binding modes of NAP1 to H2A-H2B, our group prepared the full length human NAP1 dimer, a CTAD deletion mutant dimer, and the isolated CTAD, and examined their interactions with H2A-H2B using ITC (isothermal titration calorimetry), mass spectrometry, and NMR [57]. Human NAP1 binds to H2A-H2B through two distinct modes; the CTAD binds exothermically to H2A-H2B, whereas the concave acidic surface of the NAP1 dimer core binds endothermically. Initially, the CTAD recruits H2A-H2B and subsequently transfers it to the concave surface. A second H2A-H2B heterodimer is then dynamically recruited by either CTAD [57].

Our group characterized the binding structures of one and two H2A-H2B heterodimers bound to the NAP1 dimer using integrative approaches, including NMR, SAXS, AFM (atomic force field microscopy), and MD simulations [58]. NMR and ITC analyses using several H2A-H2B amino-acids substitution mutans and N- and C-terminal halves of the CTAD revealed that the C-terminal half of the CTAD specifically binds the hydrophobic region of H2A-H2B, whereas the N-terminal half binds its basic region. The CTAD-bound structure derived from NMR was consistent with the NAP1/H2A-H2B complexes observed by AFM; in AFM measurements, the concave acidic surface of NAP1 likely binds to the mica surface, leaving the two CTAD segments available to interact with the H2A-H2B dimer.

Based on the crystal structure of the *C. elegans* NAP1 tail-less mutant bound to *C. elegans* H2A-H2B tail-less mutant [56], a model of human H2A-H2B bound to the concave acidic groove of human NAP1 was constructed. Using the CTAD-bound structure derived from NMR and the concave-bound structure derived from the crystal structure, our group generated ensemble models of NAP1 bound to one or two H2A-H2B heterodimers that fit the SAXS profiles [58]. These models were further refined by MD simulations. The resulting complex structure of NAP1 dimer bound to a single H2A-H2B heterodimer indicates an equilibrium between the CTAD-bound state (8%) and the concave-groove-bound state (92%). In the complex containing two H2A-H2B heterodimers, one heterodimer occupies the concave groove, while the second binds to the CTAD [58].

## IDRs of heterochromatin protein 1 (HP1)

**Figure 8:**
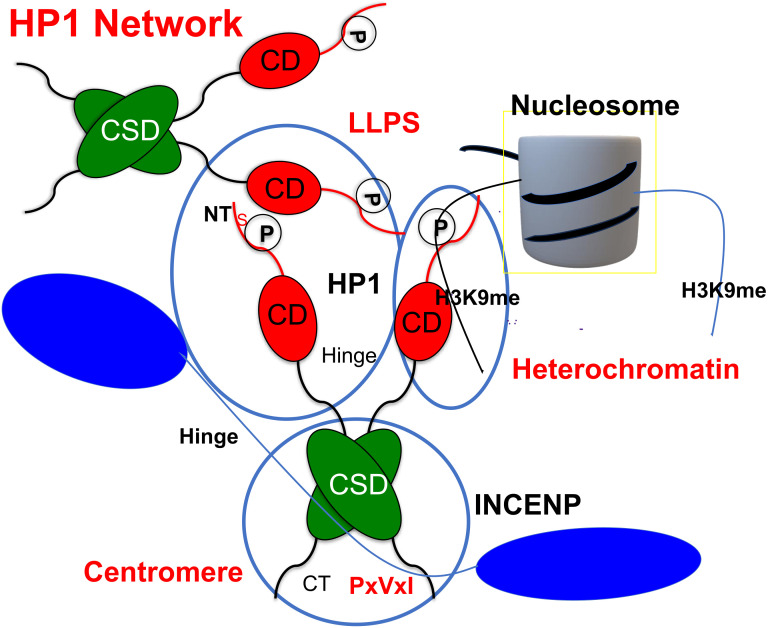
Schematic representation of HP1α interaction modes. CD captures the H3k9me via its aromatic cage and the phosphorylated N-tail (pN-tail) enhances its interaction with the H3 N-tail. The pN-tail intermolecularly interacts with CD and hinge region (HR) of neighboring HP1α to form LLPS. In addition, the hinge region of INCENP between two core domains shown by blue ellipse interacts with CSD.

To constitute B2 sub-compartments, the key factor is HP1, which has three homologues of HP1α, β, and γ [3,4]. Among them, HP1α is responsible for LLPS [59,60]. HP1α consists of an N-terminal IDR (NT), a chromodomain (CD), a hinge region IDR (HR), a chromo-shadow domain (CSD), and a C-terminal IDR (CT) (Figure 8). The CD binds to the trimethylated lysine residue of histone H3 at position 9 (H3K9me3), whereas the CSD functions as a dimerization domain and serves as a binding platform for other target proteins, including INCENP and Shugoshin, a conserved inner-centromere protein that protects centromeric cohesion [8]. HP1α uniquely contains an N-terminal IDR with four successive serine residues that are phosphorylated by CK2. Phosphorylation of the NT induces LLPS [59,60] and enhances binding to H3K9me3 [61].

### IDR-IDR interactions between the phosphorylated N-tail of HP1α and the N-tail of H3 K9me

The CD is a typical binding domain of the methylated lysine residue, using an aromatic cage typically composed of three aromatic residues of tryptophan, tyrosine and/or phenylalanine residues [62,63]. The CD of HP1α contains Tyr20, Trp41, and Phe44, which forms an aromatic cage that binds to the trimethylated lysine residue of H3K9me3 through methyl-π interactions. In addition, the H3 segment of Gln5, Thre6, Ala7, Arg8, Lys9, and Ser10 forms a β-strand that pairs with CD strand of Glu18, Glue19, Thre20, and Val21 on one side and Met 56 and Asp58 on the other. Thes aromatic cage and β-strand interactions constitute the canonical binding mode for methylated H3 tails; however, they do not account for the enhanced binding induced by phosphorylated NT (pNT).

Our group elucidated that the dynamic structures of HP1α NT-CD and pNT-CD in their free and H3K9me3-bound states using NMR, small-angle X-ray scattering (SAXS), and MD simulations [64]. SAXS revealed that pNT-CD adopts an elongated conformation with Dmax of 94 Å, compared with 79 Å for NT-CD and 56 Å for CD alone. These findings correlate well with NMR ensemble structures and MD simulations. The pNT itself adopts an extended conformation due to steric hindrance and electrostatically repulsion among the four consecutive phosphate groups. Consequently, the extended, negatively charged pNT IDR can dynamically and electrostatically interact with the basic IDR segment of H3K9me3, particularly Lys15, Arg17, and Lys18. This strongly supported by ITC measurements showing dissociation constants of 170 nM for pNT-CD, 1700 nM for NT-CD, and 13,300 nM for CD alone, demonstrating that phosphorylation of the NT is essential for the tight binding of HP1α [64].

### LLPS caused by the pNT IDR of HP1α

To investigate LLPS induced by phosphorylation of the N-terminal tail of HP1α, our group performed integrative structural analyses—including NMR, size-exclusion chromatography coupled with small-angle X-ray scattering (SEC-SAXS) and multi-angle light scattering (SEC-MALS), as well as coarse-grained molecular dynamics simulations guided by SAXS (CGMD-SAXS) [65]. NMR revealed salt-dependent chemical-shift changes in a basic segment of HR (KKKR) in HP1α, and in both the phosphorylated N-tail (pNT) and a basic segment of CD (KKYKK) in phosphorylated HP1α (pHP1α), suggesting electrostatic interactions between the extended, string-like pNT and the CD basic segment. SEC-MALS and SEC-SAXS revealed that pHP1α undergoes LLPS through dynamic multimeric aggregation, and CGMD-SAXS indicated interactions of pNT with the intra-subunit HR basic segment and the inter-subunit CD and HR basic segments. These findings suggests that intermolecular interactions between the extended pNT and these basic segments are responsible for LLPS of pHP1α [65].

Our group further found that a CSD deletion mutant (ΔCSD), containing NT, CD, and HR, also undergoes LLPS at a high concentration upon NT phosphorylation [65]. ΔCSD exhibited marked chemical shift changes in the HR basic segment, whereas phosphorylated ΔCSD (pΔCSD) showed dramatic NMR chemical shift changes in the pNT and CD basic segment. CGMD-SAXS revealed that pΔCSD exists partly as a dynamic dimer; thus, the similar chemical shift changes observed for pHP1α and pΔCSD indicate that pNT engages in inter-subunit and/or intermolecular interactions primarily with the CD basic segment. As concentration increases, pΔCSD dynamically forms dimers via interactions between pNT and the CD basic segment, and these dimers constitute the essential structural unit for LLPS [65]. Among the three mammalian HP1 homologues—HP1α, HP1β and HP1γ—only HP1α undergoes LLPS in vitro, and the CD basic segment is unique to HP1α.

### Interactions of the hinge-region IDR of INCENP with HP1 CSD

The inner centromere protein, INCENP is a core component of the chromosome passenger complex (CPC) and regulates the phosphorylation activity of Aurora B kinase, which is responsible for proper chromosome segregation [7,8]. INCENP serves as a scaffold that accommodates Survivin and Borealin/Dasra at its N-terminus and Aurora B at its C-terminus. The hinge region between these domains is a long IDR of approximately 480 amino acids and binds to HP1 through the well-known PxVxL (PVI) motif [7,66]. Recent pull-down and ITC experiments revealed that the PVI motif alone (Pro168, Val169, Val170, Glu171 and Ile172) is insufficient for binding HP1α; the C-terminal region adjacent to the PVI motif is also required [67]. NMR analysis revealed the complex structure of the HP1α CSD dimer bound to the INCENP hinge region. The sequence surrounding the PVI motif (residues 164–170) adopts a β-strand conformation, and the adjacent C-terminal region folds into an α-helix upon HP1 binding. This combined β-strand/α-helix structure has been termed the SSH domain [67].

## Closing remarks on nuclear IDRs

As described above, the IDRs of histones in NCP, nucleosome and chromatosome, as well as those of the histone chaperones FACT and NAP1 and the heterochromatin protein HP1, play essential roles in the formation of euchromatin and heterochromatin. In particular, the N-terminal tail IDR of HP1 is responsible for LLPS. Thus, histone tails also contribute to LLPS; in the crosstalk among histone tails, intramolecular interactions with core- and linker-DNA are key determinants of their modification states. In condensed nucleosome arrays, histone tails further engage in intermolecularly interactions with DNA of neighboring nucleosomes to form higher order multimers, thereby promoting LLPS [27–30].

RNA polymerase II (Pol II), which is responsible for mRNA transcription [3–6], consists of 12 subunits. Among them, Rpb1 contains a long IDR—the C-terminal domain (CTD)— composed of 52 tandem repeats of the YSPTSPS heptad sequence in humans. The CTD provides a critical binding platform for numerous factors, and its phosphorylation is essential for controlling multiple stages in transcription. Recent cryo-EM studies have revealed structural alterations of the nucleosome induced by Pol II in cooperation with FACT during transcription [17]. While these structures beautifully capture the core architectural transitions, structural information on the CTD has remained elusive.

In eukaryotes, three RNA polymerases exist: Pol I for ribosomal RNA, Pol II for mRNA, and Pol III for tRNA. Interestingly, Rpb6 is a common subunit shared among Pol I, Pol II, and Pol III. Rpb6 also contains an N-terminal IDR, which serves as a recruitment region for the general transcription factor TFIIH via the PH domain of its p62 subunit [68]. Our group determined the complex structure of the Rpb6 IDR bound to the PH domain and subsequently modeled the entire Pol II—TFIIH complex by docking the NMR structure into their respective cryo-EM maps [68,69]. The resulting model reveals a conserved binding mode shared among various TFIIH targets, including TFIIE [70], p53 [71], DP1 [72], XPC [73], and UVSSA [74], each of which uses its unique IDR to engage the PH domain.

Comparable structural work on the Rpb1 CTD will be essential for a comprehensive understanding of Pol II function. Such insights will likely require NMR studies in combination with complementary approaches, including MD simulations. Notably, the Rpb1 CTD drives LLPS, and its phase behavior is modulated by phosphorylation states and interaction with binding partners to ensure organized transcription [75]. With the advent of high-field NMR spectrometers and advanced measurements techniques, it may become possible to detect NMR signals of the Rpb1 CTD not only in isolated CTD peptides, but also within the intact Pol II complex, for example by using natural-abundance ^13^C-methyl NMR experiments to observe the threonine methyl groups present in the YSPTSPS tandem repeats. The NMR-derived dynamic properties obtained in this manner will be validated through CGMD simulations as well as SAXS.

## Conflicts of interest

The author declares no competing financial interests.

## Author contributions

The author wrote this manuscript.

## Data availability

The data analyzed during the current study are available from the corresponding author on reasonable request.

## Acknowledgments

The author would like to thank Dr. A. Furukawa for NMR experiments on the H3 and H4 N-tails of unmodified and H4-4Kac NCPs, nucleosomes, and chromatosomes, as well as on HP1 and pHP1, ΔCSD and pΔCSD, and INCENP bound to CSD; Dr. H. Ohtomo for NMR experiments on the H2A N- and C-tails and H2B N-tails of NCPs and nucleosomes, the H3 N-tails of H2Aub-NCPs and nucleosomes, the H2A-H2B heterodimer in its free and NAP1-bound states, and the NAP1 CTAD in its free state and bound to several H2A-H2B mutants; Dr. Y. Tsunaka for NMR experiments on the H3, H2B, and H2A N-tails of pAID/112-bp NCP; Dr. J. Kurita for NMR relaxation experiments; Drs. Y. Arimura and H. Kurumizaka for the initial preparation of NCPs and nucleosomes; Drs. M. Wakamori and T. Umehara for the preparation of unmodified and H4-4Kac NCPs and nucleosomes; Dr. H. Kono for MD simulations of H2A and H2B in NCP; Drs. S. Ito, C. Davidovich, and H. Koseki for the preparation and methylation experiments of H2Aub NCPs and nucleosomes; Drs. H. Ehara and S. Sekine for cryo-EM experiments of H2Aub NCP; Dr. N. Kodera for AFM measurements of NAP1 bound to H2A-H2B; Dr. T. Oda for SAXS experiments of NAP1 bound to H2A-H2B; Drs. T. Yamane and M. Ikeguchi for MD simulations of H2A-H2B and NAP1 bound to H2A-H2B; Drs. K. Sako and T. Hirota for INCENP–HP1 experiments; Drs. T. Senda and N. Shimizu for SEC-MALS and SEC-SAXS experiments of HP1, pHP1, ΔCSD, and pΔCSD; Dr. T. Terada for CGMD simulations of HP1, pHP1, ΔCSD, and pΔCSD; Drs. S. Blazquez and T. Terakawa for CGMD simulations of unmodified and H4-4Kac nucleosomes and chromatosomes; Dr. J. Nakayama for cell experiments on HP1; Drs. K. Echigoya, Y. Takizawa, and H. Kurumizaka for cryo-EM experiments of H4-4Kac chromatosomes; Dr. H. Shimojo for NMR experiments on HP1 NT-CD and pNT-CD with or without H3K9me; Dr. A. Kidera for MD simulations of NT-CD and pNT-CD with or without H3K9me; Dr. Y. Moriwaki for NMR experiments on H2A-H2B; and Dr. M. Okuda for NMR experiments on TFIIH-related proteins. This work was supported, in part, by the NMR Platform (grant no. JPMXS0450100021) from the Ministry of Education, Culture, Sports, Science and Technology (MEXT), Japan; by the Platform Project for Supporting Drug Discovery and Life Science Research (Basis for Supporting Innovative Drug Discovery and Life Science Research; BINDS) from the Japan Agency for Medical Research and Development (AMED; grant nos. JP21am0101073 and JP22ama121001); and by the Japan Society for the Promotion of Science (JSPS) Grants-in-Aid for Scientific Research (JP23H02426 and JP23K27119).
